# Early Pregnancy Diagnosis in Bovines: Current Status and Future Directions

**DOI:** 10.1155/2013/958540

**Published:** 2013-12-05

**Authors:** Ashok K. Balhara, Meenakshi Gupta, Surender Singh, Ashok K. Mohanty, Inderjeet Singh

**Affiliations:** ^1^Central Institute for Research on Buffaloes, Hisar 125001, India; ^2^LLR University of Veterinary and Animal Sciences, Hisar 125005, India; ^3^National Dairy Research Institute, Karnal 132001, India

## Abstract

An early and accurate diagnosis of reproductive dysfunctions or aberrations is crucial to better reproductive management in livestock. High reproductive efficiency is a prerequisite for high life-time production in dairy animals. Early pregnancy diagnosis is key to shorten the calving interval through early identification of open animals and their timely treatment and rebreeding so as to maintain a postpartum barren interval close to 60 days. A buffalo, the most important dairy animal in the Indian subcontinent, is known for problems related to high calving interval, late puberty, and high incidence of anestrus. Lack of reliable cow-side early pregnancy diagnosis methods further aggravates the situation. Several methods of pregnancy diagnosis are being practiced in bovine species, yet none qualifies as the ideal pregnancy diagnosis method due to the inherent limitations of sensitivity, accuracy, specificity, speed, and ease of performing the test. The advancement of molecular techniques like proteomics and their applications in animal research has given a new hope to look for pregnancy biomarker molecules in these animals. This review attempts to examine common pregnancy diagnosis methods available for dairy animals, while assessing the usefulness of the modern technologies in detecting novel pregnancy markers and designing future strategies for research in this area.

## 1. Introduction 

An early and precise pregnancy diagnosis is an important criterion for better reproductive management in livestock like cows and buffaloes. High reproductive efficiency is a prerequisite to realization of high life-time production from dairy animals. Early pregnancy diagnosis is crucial to shortening the calving interval through enabling the farmer to identify open animals so as to treat and/or rebreed them at the earliest opportunity. Ideally a 60-day postparturient barren interval in dairy animals is recommended for breeding. Dairy farmers need to recognize nonpregnancy at the earliest opportunity so as to rebreed the dam at the very next opportunity. The early embryonic period in cattle has been described to be lasting for approximately 42 days postinsemination [1], encompassing a series of events starting with fertilization and culminating in implantation (Table 1). After implantation, embryonic losses due to noninfectious causes are rare and the pregnancy becomes more secure [2, 3]. Studies on levels of progesterone, pregnancy associated glycoproteins (PAGs), interferon tau, and early pregnancy factor are some of the common clinically practised pregnancy detection methods in bovines, and each has its own benefits and limitations. Buffaloes are the most important dairy animal of the Indian subcontinent, yet they experience problems related to reproduction especially high calving interval, late puberty, and high incidence of anestrus. Lack of reliable early pregnancy diagnosis methods further aggravates the problems. Many methods of pregnancy diagnosis, both direct and indirect, are being practiced in bovine species; none till date actually qualifies as the ideal pregnancy diagnosis method due to the limitations they inherit. The advancement of molecular techniques like proteomics and their applications in animal research has opened up opportunities for research communities to look for pregnancy biomarker molecules in these animals.

In this review we have described common pregnancy diagnosis methods available for dairy animals, assessing the usefulness of the modern technologies in detecting novel pregnancy markers and designing future strategies for research in this area.

## 2. Pregnancy Detection Methods

### 2.1. Direct Method

#### 2.1.1. Per-Rectal Palpation

Cowie [5] first described transrectal palpation of the uterus as a method for pregnancy diagnosis in cattle which makes it the oldest and most widely practised method for early pregnancy diagnosis in large dairy animals even today. Traditionally, to confirm pregnancy at about day 30 of gestation onwards, the practitioners have relied on the palpation of the amniotic vesicle [6] and slipping of the chorioallantoic membranes between the thumb and forefinger [7]. In buffaloes too, palpation per rectum is a simple, economic, and the most widely practised method for pregnancy diagnosis; however, this method is only accurate from day 45 of pregnancy [8]. Though per-rectal palpation is the cheapest pregnancy diagnosis method, several studies have suggested that examining pregnant cows early in gestation by transrectal palpation increases the risk of iatrogenic embryonic mortality [9].

#### 2.1.2. Ultrasonography

By per-rectal palpation an expert can accurately diagnose an animal pregnant only after day 35 of gestation, but the application of ultrasonography has made diagnosis possible as early as day 28 after insemination [9] or even earlier [10]. The first visible changes appearing by day 21 after breeding, when fetal heartbeat can be visualized, also helped confirm a viable pregnancy [11] though it is not a routinely assessed parameter for pregnancy diagnosis. Transrectal ultrasonography has the added advantage of providing additional information on ovarian structures, identification of twins, and determination of fetal viability, age, and sex [10, 12]. Transrectal ultrasonography made a thorough examination of the reproductive health of the animal possible and, therefore, it has now become an established research tool to study bovine reproductive biology in cattle [12] and buffaloes [10]. Ultrasound is a minimally invasive, accurate, and efficient technique for early pregnancy diagnosis [13, 14] and may minimize the rare incidence of palpation-induced abortions.

Most studies on the utility of transrectal ultrasonography for pregnancy diagnosis have been conducted in cattle, but lately it has found utility in buffalo cows as well. In buffaloes, transrectal ultrasonography is most commonly used to determine pregnancy, fetal age, and sex as well as ovarian activity [15]. In early 1990s, various workers started using transrectal ultrasonography in buffaloes with visualization of the embryonic vesicle and embryo proper in pregnant buffalo cows between 19 and 22 days after AI [16]. In a field study on 260 buffaloes between 30 and 45 days after AI, sensitivity of detection of pregnancy was observed to be 97.9% [17]. However, unpublished data from researchers at the Central Institute for Research on Buffaloes, Hisar, India, suggest the accuracy for selecting pregnant buffaloes at day 21 after AI to be about 50%, which increases to almost 100% by day 30. These findings support other findings in cattle which claim that transrectal ultrasonography for pregnancy diagnosis between days 21 and 25 after breeding has sensitivity and specificity of 44.8% and 82.3%, respectively, which further increase to 97.7% and 87.7%, respectively, when conducted between 26 and 33 days after AI [18]. Similarly, Nation et al. [19] documented that the sensitivity and specificity of pregnancy diagnosis in lactating dairy cows based on ultrasonographic detection of uterine fluid as well as embryonic membranes from 28 to 35 days after AI were 96% and 97%, respectively. Direct observation of a fetus with ultrasonography was found more accurate than assays for the presence of pregnancy-specific proteins in plasma but resulted in more false negative diagnoses [20].

Per-rectal palpation and transrectal ultrasonography are direct and accurate methods for pregnancy diagnosis. Both require a great deal of skill and experience. Veterinary-grade ultrasound machines equipped with a rectal transducer are expensive in developing countries and therefore the high initial cost of this technology partly limits its practical implementation [12].

### 2.2. Indirect Method

#### 2.2.1. Progesterone

Shemesh et al. [21] proposed that the difference in peripheral plasma progesterone levels between pregnant and nonpregnant cows, 19 days after insemination, can form the basis for a very early pregnancy test. Laing and Heap [22] first documented this in milk to diagnose cows in early pregnancy. Measurement of progesterone is an indirect method for pregnancy diagnosis in many livestock species including cattle, buffaloes, sheep, and goats.

Conception extends the life of the corpus luteum (CL) by preventing the luteolytic mechanism from being triggered, thus prolonging and maintaining its functional characteristics, ensuring continued high progesterone levels [31]. Progesterone maintains the uterine endometrium in a state which supports embryonic development, implantation, and foetoplacental development. Progesterone concentrations vary with the stage of the estrous cycle which makes it one of the most commonly studied reproductive hormones in bovine ruminants for pregnancy detection and ovarian activity [32].

Studies in the bovine estrous cycle indicate that the milk or serum progesterone concentrations reach a maximum value 13-14 days after estrus, and if the animal is pregnant, these continue to remain elevated up to day 21 after fertilization [33] and beyond. These high levels of progesterone in serum or milk between days 18 and 24 after insemination form the basis of establishment of pregnancy in cattle [34, 35]. Interferon-*τ* exerts its antiluteolytic effect by inhibiting the endometrial expression of oxytocin receptors, through which oxytocin stimulates pulsatile PGF_2*α*_ release [36]. Although low progesterone concentrations at 18 to 24 days after breeding can accurately predict nonpregnancy, high progesterone concentrations during this period are not the specific indicators of pregnancy due to variations among cows in duration of the estrous cycle as well as the incidence of early or late embryonic mortality. The advantages of progesterone assay for pregnancy diagnosis include noninvasive collection of milk sample and the feasibility to conduct the test on the farm using commercial cow-side milk progesterone test kits [28, 37–39], though the sensitivity gets compromised to some extent with these assay kits.  Table 2 describes the work in different labs on the level of progesterone in pregnant and nonpregnant bovines.

In buffalo cows, it is quite evident that the progesterone levels in milk are four to five times higher than those in blood plasma [25, 26]. Just like cattle, buffaloes too can be accurately diagnosed as nonpregnant by determination of plasma progesterone concentrations 21 days after insemination [27]. A major constraint in using progesterone assay for pregnancy diagnosis is its use only in cases where AI or breeding dates are known/recorded and not randomly in the herd. Nevertheless, progesterone analysis remains the most common clinical use of any of the reproductive hormones.

#### 2.2.2. Estrone Sulphate

Estrone sulphate is a conjugated steroid product of estrone, present predominantly in the bovine placentomes [40] and it is the major estrone present in the fetal (allantoic and amniotic) fluids and maternal peripheral plasma of cows with measurable quantities detectable by day 52 onwards till the end of gestation [41]. Its concentrations increase from day 60 and plateau around day 150 after insemination [42]. However, reliable pregnancy detection is possible only after day 100 of gestation and therefore this test can only detect late pregnancy [43]. Concentration of estrone sulphate in the maternal body fluids is a useful indicator for the placental functions especially those related to embryonic growth [44]. In zebu and crossbred cattle and Murrah buffaloes, Prakash and Madan [45] reported below detection levels (<50 pg/mL) of estrone sulphate during the first two months, followed by sharp increase in the fourth month and values stabilized after reaching the highest levels in the sixth month of pregnancy. Levels of estrone sulphate in different maternal body fluids, namely, milk and blood plasma, can be utilised as the criteria for confirming pregnancy by after 110 day insemination in bovine species [46]. Estrone sulphate concentrations have also been frequently correlated to fetal numbers [44], as these are higher when the number of developing foetuses is more than one. Yet, estrone sulphate is not an ideal pregnancy biomarker as the plasma and milk profiles are influenced by many other factors such as genetic makeup, weight, parity status, and environment [46].

#### 2.2.3. Conceptus and Placenta Secreted Products

The very fact that pregnancy brings about numerous physiological changes in the female body through secretion/altered secretion of various biomolecules, which often are proteins or their metabolites, supports research endeavours aimed at identifying novel proteins as the candidate molecules for pregnancy detection. Human chorionic gonadotropin (hCG), discovered for the first time by Aschheim and Zondek [47] in the urine of pregnant women in 1927, is perhaps the best example of a placental protein hormone used for pregnancy diagnosis. With the advancement of biotechnological tools, hCG based pregnancy diagnosis has become the simplest, cheapest, and most commonly practised test for humans to diagnose pregnancy as early as 8–10 days after conception [48]. Homologous to the human protein, only higher primates produce a chorionic gonadotropin (CG) for maintaining luteal activity during early pregnancy, while ruminants produce type I interferon as an antiluteolytic factor during this period [49].

#### 2.2.4. Early Conception Factor (ECF)

Early pregnancy factor (EPF, also known as early conception factor—ECF)—a 10.84 kDa protein [50], is present in the sera of pregnant mammalian females, detectable within 6 to 24 hours of fertilization [51] and disappearing within 24 to 48 after death or removal of the embryo [52], EPF is present in the serum up to two-thirds of the gestation [53]. EPF remains the earliest serum benchmark for positive fertilization and hence successful conception. This novel pregnancy-specific protein has high immunosuppressive ability which is demonstrated by rosette inhibition test, a bioassay first demonstrated in pregnant mice [54]. Laleh et al. [55] demonstrated significant differences in rosette inhibition titres of pregnant and open cows with values being 8–10 and 3–5, respectively.

EPF is reported to be present in the pregnant sera of most mammalian species including humans [56, 57], mice [51], sheep [58, 59], cows [60–62], pigs [63], mares [64], and some wild animals [65]. In buffalo pregnancy, Chander [66] demonstrated decreased E-rosette formation but failed to demonstrate the presence of a rosette inhibiting factor (RIF, which probably would have been EPF) in the serum. Antibodies raised against a cow serum glycoprotein were used to detect EPF [67] leading to development of a lab method, which has been commercialized in the USA as Early Conception Factor (ECF) test (Concepto Diagnostics, Knoxville, TN) claiming detection within 48 hours. Extensive study on the effectiveness of the commercial ECF test for diagnosing nonpregnancy revealed a high degree of nonreliability of the test wherein only 44.4% and 55.6% of the confirmed nonpregnant heifers were identified correctly by serum ECF analysis at days 1 to 3 and days 7 to 9 after AI, respectively [61]. Similar conclusions were drawn by [60] and [62]. Although EPF is secreted in early pregnancy, it is not strictly pregnancy specific because of its secretion from nonplacental sources such as tumors and transformed cell lines [50], which makes it an erroneous pregnancy detection method. EPF belongs to a family of heat shock proteins, though detected extracellularly and having immunosuppressive and growth factor properties [68]. These properties are crucial to avoid rejection of an antigenically alien embryo and support its development. Therefore, with the advent of modern biosciences, there is hope that these changes could be identified and used as diagnostics for very early detection of pregnancy. However, the practicability of such an early test may still remain low due to high incidence of losses during the first 15 days of conception [2].

#### 2.2.5. Interferon-*τ* (IFN-*τ*)

Moor and Rowson [69], the pioneers of sheep embryo transfer, transferred embryos on days 12, 13, and 14 to unmated ewes and suggested interactions between the embryo and uterus that influence the luteal function and result in establishment of pregnancy. Godkin et al. [70] purified ovine trophoblastic protein-1 (oTP-1), an early secretory protein of the sheep blastocyst, from *in vitro* cultured days 14–16 conceptuses. They revealed that oTP1 acts on the maternal endometrium thereby eliciting maternal responses which contribute to the maintenance of pregnancy. Imakawa et al. [49] reported the primary amino acid sequence of oTP-1 to demonstrate that the protein is most probably an interferon-alpha. Later research proved that the secretions from the conceptus are, in fact, responsible for the maternal recognition of pregnancy [71, 72].

Interferon-*τ*, a novel type I interferon [73], is first produced by the conceptus between days 12-13 after insemination in sheep and days 14–16 in cattle [74–76]. High ovine IFN-*τ* levels are attained on days 12-13 before luteolysis could actually be triggered [77]. In ruminants, IFN-*τ*, a 172 amino acid polypeptide [73], blocks transcription of estrogen receptor alpha and oxytocin receptors in endometrial cells [78], while downregulating the expression of enzymes cyclooxygenase-2 and prostaglandin F synthase [79], thus preventing PGF release necessary for luteolysis.

IFN-*τ*, acting within the uterine cavity [80] with extremely low levels in extrauterine tissues and peripheral circulation, prevents direct use of IFN-*τ* as an early pregnancy diagnosis molecule [81]. Rapid advancement of molecular techniques in the last two decades has opened new avenues for exploring this unique molecule as a pregnancy marker for ruminants through studies on IFN-*τ* stimulated genes (ISG), namely, interferon-stimulated protein 15 kDa (Isg15), myxovirus resistance 2 (MX2), and 2′-5′ oligoadenylate synthetase (OAS1), in peripheral blood leukocytes during early pregnancy [82, 83]. Microarray analysis further indicated that many genes, including IFN-*τ* stimulated, are upregulated during early pregnancy [84–86]. Green et al. [84] have however shown that the differential expression of such genes is influenced by the parity of the animal, being more definite in heifers as compared to multiparous animals. All these experiments have suggested IFN-*τ* stimulated genes to be potential pregnancy detection biomarkers; still there is no field level test available based on these markers.

#### 2.2.6. Pregnancy Associated Glycoproteins (PAGs)

Relocation of the extra embryonic trophoblastic cell layers to the endometrium [87] between days 20 to 28 and secretions from the conceptus lead to successful implantation and continuation of pregnancy in ruminant species [88]. The pregnancy associated glycoproteins (PAGs) are secretory products from the mono- and binucleated trophoblastic cells in bovine placentomes [89]. Among these glycoproteins, Butler et al. [90] detected two pregnancy-specific proteins in the sera of pregnant cows, a 65–70 kDa and a 47–53 kDa protein at pI 4.6–4.8 and 4.0–4.4, respectively. Of these, the former showed an immune reaction similar to that of *α*_1_-fetoprotein, while the latter showed no reactivity with known proteins and it was given the name “protein B” or the “pregnancy-specific protein B” (PSPB) in bovines. Further purification and characterization of several isoforms from bovine foetal cotyledons found that protein B is actually a 67 kDa PAG [91]. Biochemical and functional investigations established these proteins to be enzymatically inactive members of the aspartic proteinase superfamily having homology to pepsin, chymosin, cathepsins D, and enzyme renin [92, 93]. PAGs are a very complex group of proteins, a fact proven by the already documented 22 distinct cDNA libraries [94]. The three most studied bovine PAGs PSPB, PAG 67 kDa or bPAG-1 [93], and PSP60 [95] are isomers of the same protein having similar N-terminal sequences [96]. Transcription of bPAG-2 and -11 mRNA is seen all through the pregnancy; -4, -5, and -9 mRNAs in early pregnancy and bPAG-1 mRNA are detectable only after day 45 [97]. Interestingly, bovine PAG-4 and bPAG-1 mRNA are highly transcribed till day 250 of gestation but become indiscernible at the end [97]. The six N-glycosylation sites [98] are responsible for the variations in molecular weight and half-life of PAGs [99] and is also the reason for expression of different PAGs during different stages of gestation [97, 100].

Very recently, it has been observed that placental defects, commonly seen during somatic nuclear transfers in cattle, are complemented by unusually high plasma levels of PAGs, probably due to diminished clearance of these proteins following changes in the glycosylation patterns [94]. PSPB is detectable in the serum of pregnant cows over a long period of gestation starting at about the fourth week [101] of gestation to several weeks after parturition [102]. High circulating levels of these proteins on days 80 to 100 postpartum restrict their use as a pregnancy diagnosis test, except in heifers [102, 103].

Sasser and coworkers [104] developed double antibody radioimmunoassay for the serological detection of PSPB for pregnancy detection in cattle and found serum levels increasing progressively from 1 ng/mL after day 30 to 9 ng/mL, 35 ng/mL, and 150 ng/mL after three, six, and nine months of pregnancy, respectively. The study claimed PSPB detection to be more accurate than the traditional rectal palpation method for pregnancy detection. Green et al. [105] developed a sandwich ELISA, using anti-PAG monoclonal antibodies, which were able to detect PAG in all pregnant animals with concentrations of 8.75 ng/mL on day 28, the highest at 588.9 ng/mL during the week of parturition, and very low levels within 4 weeks postpartum. Silva et al. [106] predicted 93.7, 95.4, and 96.2% accuracies for first, second, and third postpartum timed artificial inseminations, which were in agreement with other commonly practiced pregnancy detection methods. Different homologous (RIA-497) and heterologous radioimmunoassay systems (RIA-706, RIA-780, RIA-809, and RIA-Pool) developed for measurement of ruminant blood PAG concentrations are highly correlated and can be used for pregnancy detection of 30–80 days [107]. Radioimmunoassay of pregnant sera of zebu cattle established PAG concentrations to be 6.0 ng/mL, 196.0 ng/mL, 1095.6 ng/mL, and 348.4 ng/mL at 8 weeks, at 35 weeks, at term, and at 2 weeks postpartum, respectively, a pattern similar to other breeds of cattle [108]. Results of PAG-RIA based pregnancy diagnosis in buffaloes have also been encouraging with a high degree of accuracy of diagnosis as early as day 31 with 100% sensitivity and 90–100% specificity [109]. PAGs are used for development of bench-top pregnancy detection methods [110], which are now commercially available as BioPRYN (BioTracking, Russia), DG29 (Genex Cooperative Inc., USA), and IDEXX (IDEXX Laboratories, Inc., USA). BioPRYN blood test is the most extensively used PAG based kit for pregnancy detection in ruminants. By May 2010, already there were 2 million cattle blood tests conducted for pregnancy detection (http://biotrackingcom.siteprotect.net/about/timeline).

## 3. Current Research in Biomarkers for Pregnancy

It is presumed that the monitoring of sequential changes in blood proteome profile from the day of estrus to successful conception and through progression of gestation can lead to discovery of molecules, which will perhaps be novel and specific to the physiological stage of the animal. In order to qualify as a marker for pregnancy, the candidate molecule should be able to accurately determine the pregnancy status as early as possible with minimum false positives or false negatives. Additionally, the biological marker for pregnancy should have the following desired characteristics:

- specifically upregulated or downregulated during pregnancy,
- least affected by nonanimal factors like feed, environment, and drug interactions,
- having the ability to reflect age as well as viability of the conceptus,
- present in easily accessible body fluids like serum, milk, urine, and vaginal discharge,
- expressed over a considerable period of time to give ample time for diagnosis,
- revealing the result immediately.

Proteomics [111] is large scale study of protein functions, protein expression, protein-protein interactions, or posttranslational modifications in a particular cell, tissue, or organism and is intended for identification of all the proteins present. Proteomics provides an opportunity to simultaneously analyse thousands of proteins in a single experiment from a complex mixture of proteins in various body fluids [112]. This will help in identifying specific and sensitive biomarkers fulfilling the characteristics of uniqueness for a pregnancy diagnosis molecule. Main objectives of the proteomics research include documentation of biomarkers, altered protein expression patterns indicative of pathophysiological changes, and therapeutically important drug targets [113]. Easily reachable body fluids like blood serum and milk have a wide range of abundant proteins and these few proteins make up about 97% of the total serum and milk proteome and thereby interfere in the proteomic analysis (reviewed by [113]). Conversely, it is the low abundance proteins which have the highest prospect of being the novel biomarkers of changes in internal milieu of body. To sort the problem of high abundance proteins, two approaches are suggested: removal of abundant proteins (usually by immunoaffinity) and concentration of the low abundance/scarce proteins with simultaneous removal of high abundance proteins, technically known as combinatorial peptide ligand libraries, CPLL [114]. Commercially available *ProteoMiner* kit from M/s BioRad is CPLL based. Both approaches, however, lead to loss of a significant portion of the low abundance proteins along the high abundance proteins, yet the later approach is preferred [114].

There is limited information on the bovine proteome in relation to pregnancy. Jin et al. [115] performed proteomics analysis using blood serum samples of pregnant and nonpregnant Holstein dairy cattle at 21 and 35 days after AI and reported composite profiles of key proteins involved in early pregnancy and suggested the potential use of identified proteins to detect early pregnancy in bovines. These included nine pregnancy-specific spots in day 21 and day 35 serum samples. Pregnancy-specific proteins were identified as transferrin, albumin, IgG2a heavy chain constant region, and immunoglobulin gamma heavy chain variable region. Further, differential proteomic analysis of milk samples from pregnant and nonpregnant cows revealed 16 protein spots, 14 pregnancy specific and 2 spots downregulated in the pregnant milk sample [116]. Pregnancy-specific proteins were identified as serum albumin precursor, IgG1 heavy chain constant region, conglutinin precursor, epithelial keratin 10, and kelch-like ECH-associated protein. Though some identified spots were abundant milk or serum proteins, their molecular weights and pI values were different from main milk or serum proteins. This may suggest that these proteins could be pregnancy-specific subunits or fragments of albumin and IgG or carrying differentially expressed small proteins, which may ultimately have potential for pregnancy detection.

Encouragingly enough, these studies need further investigations for arriving at some sort of pregnancy detection method. Preliminary studies in buffalo cows on blood proteome too detected significant changes in many proteins in 2DE gels [117]. Important proteins found on MS analysis of these were synaptojanin-1, apolipoprotein A-1, apolipoprotein B, Keratin 10, and Von Willebrand factors, which are documented to have a role in embryogenesis and early pregnancy.

Data generated out of sequential blood proteome analysis during pregnancy can have several other applications as well, for example, studying fetal viability, genetic disorders, and so forth. Trisomy 21 pregnancies can be detected with high accuracy by maternal serum proteomic analysis in humans [118]. In the absence of a single indicator for a particular life process, a combination of the expression patterns of more than one substance can be used for a purposeful analysis as in the quadruple test where levels of four blood constituents (alpha-fetoprotein, human chorionic gonadotropin, unconjugated oestriol, and inhibin-A) have been used to predict the probability of Down's syndrome in babies [119]. A pregnancy detection test on the same lines as the human quadruple test can be tried where instead of one we can consider protein profiles of more proteins. This approach too will require a thorough analysis of the bovine proteome, before such a test with high accuracy is available to the livestock owners.

## 4. Conclusion

Early pregnancy diagnosis is an important aspect for optimizing dairy production, yet none of the present day methods qualifies as an ideal diagnostic due to limitations of accuracy, later stages of applicability, and requirement for elaborate instrumentation and laboratory setup. This warrants further research on developing novel early pregnancy diagnostics for livestock species. Currently available state-of-the-art instrumentation and proteomics techniques instil hope for finding molecules—exclusively related to intricate maternal metabolic alterations necessary to align with physiology of early embryonic development and its signalling for maternal recognition of pregnancy and continued survival. Though these techniques are still in their infancy in animal science research, they hold great promise to address a long-awaited breakthrough in pregnancy diagnosis in livestock.

## Figures and Tables

**Table 1:** Important events during the early embryonic period.

Days of pregnancy	Event
Days 0-1	Fertilization, single-cell embryo (zygote) in oviduct
Day 2	Early cleavages in the oviduct (up to 8 cell stages), activation of embryonic genome
Days 3-4	Embryo enters the uterus
Days 5-6	16–32 cell zona-enclosed embryo progressing into compact morula stage
Days 7-8	Formation of a blastocoele with differentiation of embryonic cells
Days 9-10	Blastocyst expansion and hatching from the zona pellucid
Days 11–15	Blastocyst elongation from a tubular to a filamentous structure
Days 14–19	Maternal recognition of pregnancy
Days 19-20	Implantation begins
Day 21	Caruncles-cotyledons appear
Days 22–41	Implantation progresses
Day 42	Implantation completed

Compiled from available information (Morris and Diskin, 2008 [3]; Hafez, 1993 [4]).

**Table 2:** Progesterone levels in different sample types in bovine species.

Bovine species	Sample type	Day after insemination	P4 conc. (ng/mL)		Reference
			Pregnant (ng/mL)	Nonpregnant (ng/mL)	
Cow	Milk	(i) 0 or 1, (ii) 9 or 10, (iii) 21 or 22, (iv) 27 or 28	1.5, 11.1, 12.0, 12.5	1.2, 10.3, 3.0, 6.8	Zaied et al., 1979 [23]
Cow	Milk	18	>8	—	Simersky et al., 2007 [24]
Buffalo	Milk, plasma	21–35 each	16.01 and 3.61, respectively	0.41–2.67	Kamboj and Prakash, 1993 [25]
Buffalo	Plasma	0, 13	0.1 and 3.6, respectively	0.6 (18th day of cycle)	Batra et al., 1979 [26]
Buffalo	Plasma	21 or 22	1.0	<0.7	Perera et al., 1980 [27]
Cow	Milk	20	>11	<8	Pennington et al., 1985 [28]
Buffalo	Milk	18–22	24.83	2.89	Singh and Puthiyandy, 1980 [29]
Cow	Faeces	18–24	>50 as compared to nonpregnant	—	Isobe et al., 2005 [30]
